# Supplementary material for: MK2/p38/p53 Suppress Basal IL-1β and Non-Canonical NF-κB Signaling in Macrophages
Source: Int J Mol Sci. 2026 Apr 2;27(7):3232. doi: 10.3390/ijms27073232 (PMC13072840; doi:10.3390/ijms27073232)
Supplement: Supplementary file 1 [file ijms-27-03232-s001.zip › ijms-4166638-supplementary.pdf]

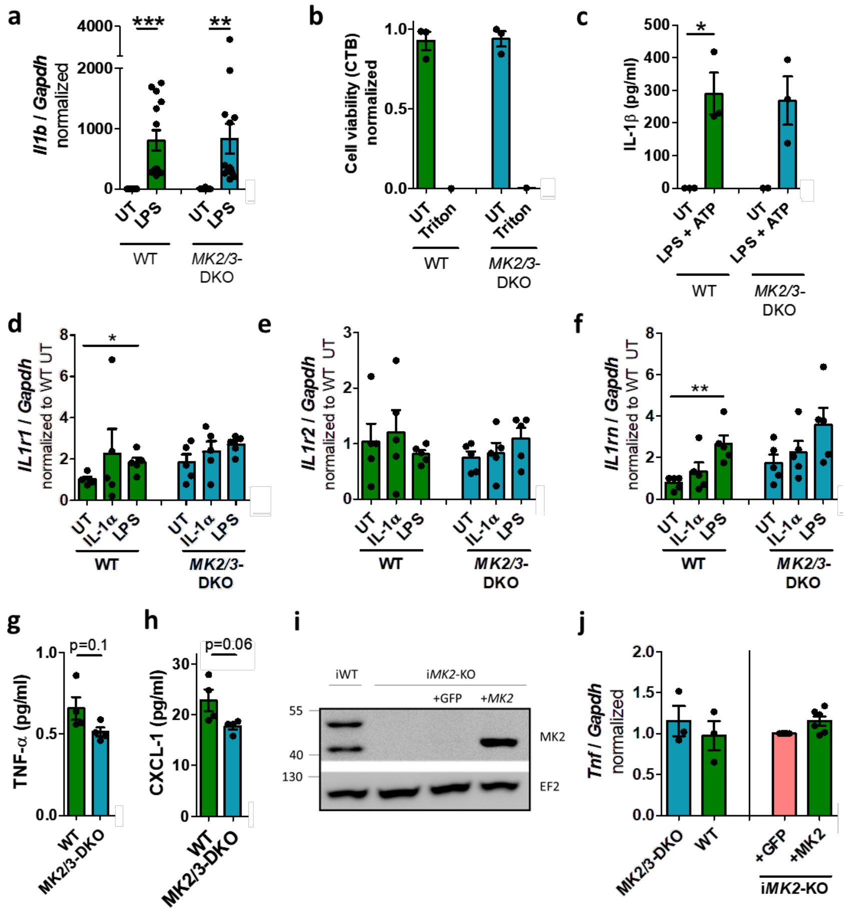


**Supplementary Figure S1.** (a) LPS-treated (100 ng/ml, 1h) wild type (WT) and *MK2/3*-double-knockout (DKO) bone marrow-derived macrophages (BMDMs) have comparable *Il1b* mRNA levels. WT *n*=14, DKO *n*=13. (b) Normalized cell viability of resting WT and *MK2/3*-DKO BMDMs was measured using a CellTiter-Blue (CTB) assay. (c) The IL-1β concentration in the supernatant of LPS-treated (1 μg/ml, 2h) *MK2/3*-DKO BMDM is similar to that of WT BMDM after the addition of ATP (3 mM, 8h). The mRNA levels of the IL-1 receptors (d) *IL1r1*, (e) *IL1r2*, and (f) *IL1rn* are similar in WT and *MK2/3*-DKO BMDMs. The cells are either untreated (UT), IL-1*α* (5 ng/ml, 1h) or LPS (100 ng/ml, 1h)-treated. Basal levels of (g) Tumor necrosis factor (TNF)-*α* and (h) chemokine C-X-C motif ligand-1 (CXCL 1) are not elevated in the serum of *MK2/3*-DKO mice compared to WT mice. *n*=6 mice/group, whereby one sample/group was pooled from 3 mouse sera. (i) Western Blot of immortalized WT BMDMs (iWT), immortalized *MK2*-KO BMDMs (i*MK2*-KO), and i*MK2*-KO transduced with either an empty vector as control (i*MK2*-KO +*GFP*) or rescued with MK2 (i*MK2*-KO +*MK2*). (j) *Tnf* mRNA is neither affected in resting BMDM (left) nor i*MK2*-KO cells (right). Mean ± SEM, Student's t-test, * $p < 0.05$, ** $p < 0.01$, *** $p < 0.001$.

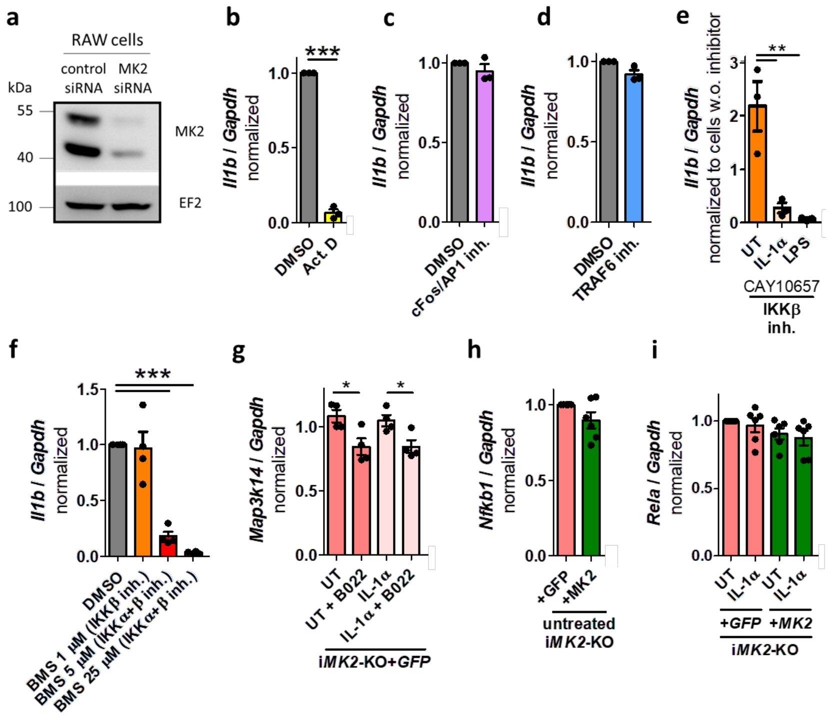


**Supplementary Figure S2.** (a) Western Blot of RAW cells treated either with control or *MK2* siRNA. *Il1b* mRNA levels in i*MK2*-KO +*GFP* cells after treatment with (b) Actinomycin D (Act. D) (10 µg/ml, 2h), (c) the cFos/AP1 inhibitor T-5224 (20 µM, 2h), (d) the SML1160 CD40-TRAF6 inhibitor (1 µM, 2h), (e) the IKKβ inhibitor CAY10657 (10 µM, 2h); untreated (UT), IL-1*α* (5ng/ml, 1h), or LPS (100 ng/ml, 1h) treated, and (f) IKK inhibitor BMS-345541 (2h). (g) *Map3k14* mRNA level after treatment with B022 inhibitor (5 µM, 2h), (h) *Nfkb1*, and (i) *Rela* mRNA levels in UT- or IL-1*α* (5ng/ml, 1h)-treated i*MK2*-KO +*GFP* cells compared to i*MK2* KO +*MK2* cells. (b-d, g-i) Student's t-test, (e) 1W-ANOVA with Tukey's Multiple Comparison Test, (f) 1W-ANOVA with Dunnet's Multiple comparison Test, Mean ± SEM, * $p < 0.05$, *** $p < 0.001$.

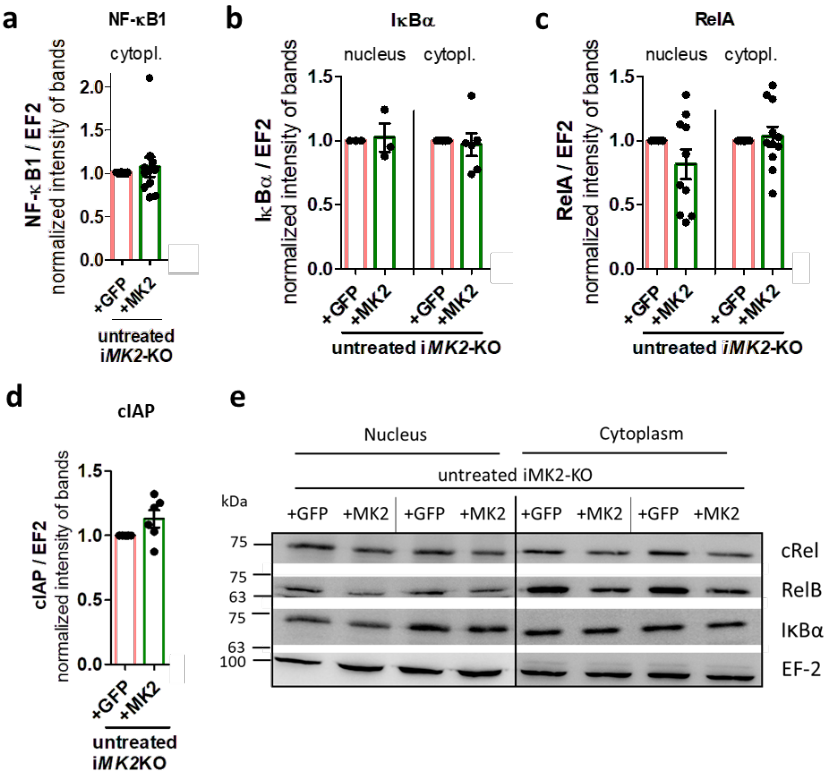


**Supplementary Figure S3.** (a) Normalized western blot band intensities of NF-κB1, (b) IκB*α* and (c) RelA in the nucleus and cytoplasm of untreated i*MK2*-KO +*GFP* and i*MK2*-KO +*MK2* macrophages. (d) Normalized western blot band intensities of basal cIAP of i*MK2*-KO +*GFP* and +*MK2* cells. (e) One representative Western Blot showing two independent experiments regarding the protein levels of cRel, RelB, IκB*α* and EF2 (used as a control) in the nuclear and cytoplasmic fractions of untreated i*MK2*-KO +*GFP* and +*MK2* cells. Student's t-test, Mean ± SEM.

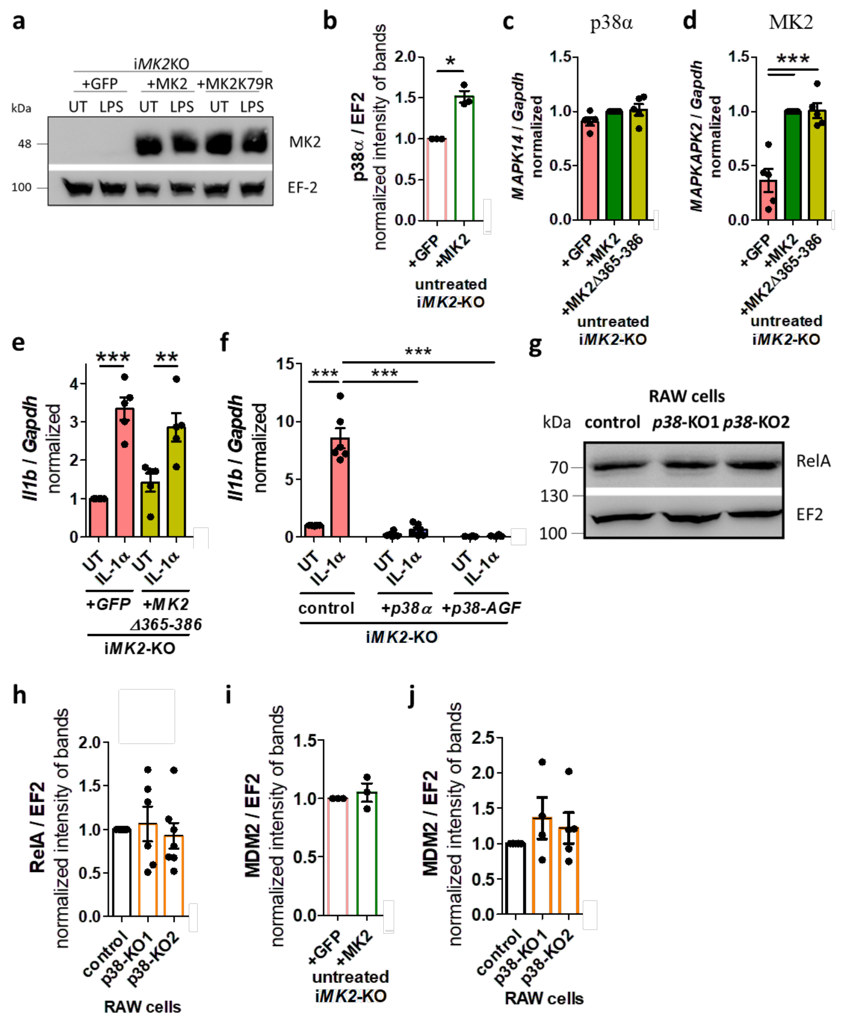


**Supplementary Figure S4.** (a) Rescued *MK2* and *MK2K79R* in i*MK2*-KO cells have comparable protein level. (b) Normalized Western Blot band intensities of p38*α* in untreated i*MK2*-KO +*GFP* and +*MK2* cells. (c) *MAPK14* and (d) *MAPKAPK2* mRNA levels in i*MK2*-KO +*GFP*, +*MK2*, and +*MK2-Δ365-386* cells. (e) MK2-Δ365-386 does not affect the *Il1b* mRNA levels in UT or IL-1*α*-treated cells. (f) *Il1b* mRNA is reduced in IL-1*α* (5 ng/ml, 1h)-treated i*MK2*-KO +*p38α* and kinase-inactive mutant +*p38-AGF* macrophages compared to control cells. (g-h) The RelA protein level is similar in *p38α*-KO cells compared to control RAW 264.1 cells. (i) MDM2 protein levels are similar in i*MK2*-KO +*GFP* and +*MK2* macrophages, as well as in (j) *p38α*-KO RAW 264.1 cells. (b, i) Student's t-test, (c-j) 1W-ANOVA followed by Tukey's Multiple Comparison Test, Mean ± SEM, * $p < 0.05$, ** $p < 0.01$, *** $p < 0.001$.

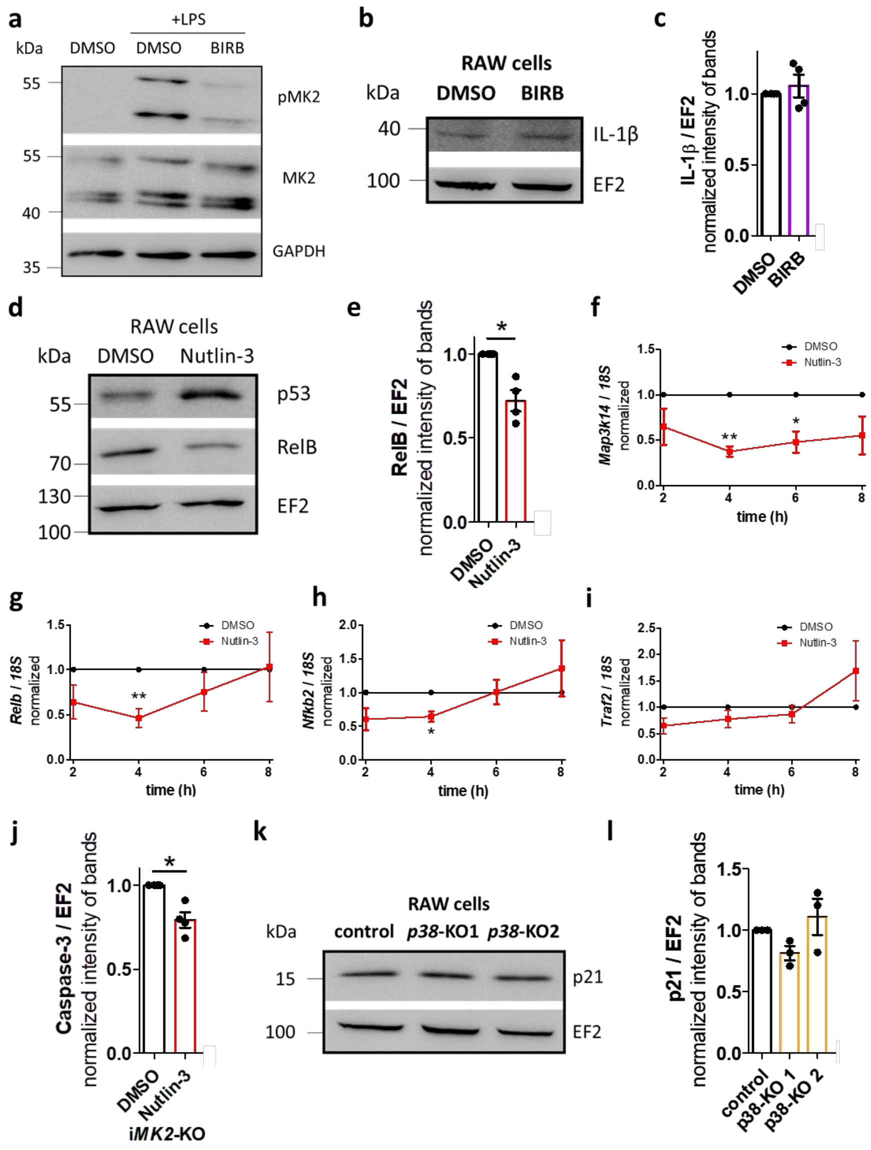


**Supplementary Figure S5.** (a) p38 inhibitor BIRB 796 (1 μM, 5h) inhibits phospho-MK2 (pMK2) in LPS-treated (100 nM, 30min) RAW 264.1 cells. One representative Western Blot of *n*=2. (b-c) BIRB796 (1 μM, 5h) has no effect on the level of IL-1β protein in the cytoplasmic fraction of resting RAW 264.1 cells. (d-e) Nutlin-3 treated (20 μM, 8h) RAW 264.1 macrophages show increased p53 and reduced RelB protein levels. (f) *Map3k14* (*n*=4), (g) *Relb* (*n*=5), (h) *NFkb2* (*n*=4) and (i) *Traf2* (*n*=5) mRNA level of Nutlin-3 (20 μM) treated i*MK2*-KO cells. (j) Nutlin-3 (20 μM, 4h) treated i*MK2*-KO cells have reduced levels of full-length Pro-caspase-3 protein. (k-l) p21 protein level in the nuclear fraction of resting *p38*-KO and control RAW 264.1 cells. (e, j) Student's t-test, (f-i) 2W RM-ANOVA with Bonferroni posttests, mean ± SEM, * $p < 0.05$, ** $p < 0.01$.
